# Microbial Communities Influence Soil Dissolved Organic Carbon Concentration by Altering Metabolite Composition

**DOI:** 10.3389/fmicb.2021.799014

**Published:** 2022-01-20

**Authors:** Tayte P. Campbell, Danielle E. M. Ulrich, Jason Toyoda, Jaron Thompson, Brian Munsky, Michaeline B. N. Albright, Vanessa L. Bailey, Malak M. Tfaily, John Dunbar

**Affiliations:** ^1^Biological Sciences Division, Pacific Northwest National Laboratory, Richland, WA, United States; ^2^Bioscience Division, Los Alamos National Laboratory, Los Alamos, NM, United States; ^3^Environmental Molecular Sciences Laboratory, Pacific Northwest National Laboratory, Richland, WA, United States; ^4^Department of Chemical and Biological Engineering, Colorado State University, Fort Collins, CO, United States; ^5^Department of Environmental Science, The University of Arizona, Tucson, AZ, United States

**Keywords:** DOC, microbial communities, FTICR mass spectrometry, metabolites, bacteria, fungi

## Abstract

Rapid microbial growth in the early phase of plant litter decomposition is viewed as an important component of soil organic matter (SOM) formation. However, the microbial taxa and chemical substrates that correlate with carbon storage are not well resolved. The complexity of microbial communities and diverse substrate chemistries that occur in natural soils make it difficult to identify links between community membership and decomposition processes in the soil environment. To identify potential relationships between microbes, soil organic matter, and their impact on carbon storage, we used sand microcosms to control for external environmental factors such as changes in temperature and moisture as well as the variability in available carbon that exist in soil cores. Using Fourier transform ion cyclotron resonance mass spectrometry (FTICR-MS) on microcosm samples from early phase litter decomposition, we found that protein- and tannin-like compounds exhibited the strongest correlation to dissolved organic carbon (DOC) concentration. Proteins correlated positively with DOC concentration, while tannins correlated negatively with DOC. Through random forest, neural network, and indicator species analyses, we identified 42 bacterial and 9 fungal taxa associated with DOC concentration. The majority of bacterial taxa (26 out of 42 taxa) belonged to the phylum Proteobacteria while all fungal taxa belonged to the phylum Ascomycota. Additionally, we identified significant connections between microorganisms and protein-like compounds and found that most taxa (12/14) correlated negatively with proteins indicating that microbial consumption of proteins is likely a significant driver of DOC concentration. This research links DOC concentration with microbial production and/or decomposition of specific metabolites to improve our understanding of microbial metabolism and carbon persistence.

## Introduction

Through the decomposition process, carbon (C) is either released as CO_2_ by microbial respiration, with important feedbacks to global warming, or retained in soil contributing to carbon storage and soil nutrient retention (Schimel and Schaeffer, 2012; Trivedi et al., 2013; Lehmann and Kleber, 2015). The amount of C stored in the soil matrix is a function of the physical structure of the soil, chemical compounds in the soil, and metabolism by the soil microbial community (Krull et al., 2001). Soil physical structure coupled with the types of chemical compounds alter the accessibility of soil carbon by governing either the physical surface area available for interaction or through chemical sorption of carbon molecules to minerals (Schimel and Schaeffer, 2012; Newcomb et al., 2017). The microbial drivers of carbon fate are strongly influenced by environmental factors that vary widely by soil depth, location, and season that alter microbial composition and function (Torsvik and Øvreås, 2002; Berg and Smalla, 2009; Fierer, 2017). Simplified microcosms that control for environmental factors provides a direct way to identify the impact of microbial metabolism in the soil (Albright et al., 2020). Further, several experiments have been conducted to determine the rate and mechanisms by which added substrates are initially decomposed (Bailey et al., 2006) but less understood is the way that compounds or compound classes within complex mixtures correlate with carbon storage (Smith et al., 2007).

The decomposition process results in a pool of degradation byproducts composed of plant and microbial compounds. Extracellular enzymes produced by both bacteria and fungi (Medie et al., 2012; Baldrian, 2017) play a role in degrading litter and producing consumable forms of carbon necessary for microbial growth and activity (Caldwell, 2005; Rillig et al., 2007). Further, microbial turnover contributes necromass to dissolved organic carbon (DOC) composition with various taxa releasing a range of microbial products including hydrophobins, melanin, chitin, β-glucans, glycoproteins, lipids, etc (Trigo and Ball, 1994; Fernandez and Koide, 2012; Siletti et al., 2017). The chemical composition of DOC produced during decomposition may affect the amount of carbon ultimately retained in the soil, with certain compounds associated with increased carbon storage either via their affinity for soil adsorption or through their influence on microbial community metabolism. For example, Kallenbach et al. (2016) identified microbe-derived proteins and lipids as being positively associated with increased soil organic matter potentially due to the presence of carboxyl groups that bind to soil. Identifying these compounds and their relationship with microorganisms will provide key insights to mechanisms of carbon storage.

The objective of this study was to determine if the accumulation of DOC can be related to the chemical carbon profile generated by the decomposing substrate and soil microbes; that is, *are certain compounds and compound classes in DOC predictive of greater concentrations of soil DOC and carbon storage potential?* In the current study, we examine links between the community composition of microbes, and chemical composition and concentration of DOC. We extend the work of Albright et al. (2020); they performed a common garden microcosm experiment where 206 soil microbial communities were inoculated on a common substrate of *Ponderosa pine* litter and sand in homogeneous laboratory microcosms. Cumulative DOC was measured after 44 days of decomposition and varied by 5-fold across all microcosms. Microcosm studies allow for a more reproducible environment where microbial interactions with metabolites can be isolated from many confounding factors. They can be used to inform models and new hypotheses that can then be tested in more complex systems. In this study, we characterized the chemical composition of a subset of samples collected from the high and low extremes of the DOC concentration distribution from the Albright et al. (2020) study. We used Fourier transform ion cyclotron resonance mass spectrometry (FTICR-MS) and applied machine learning and indicator species approaches to the previously identified bacterial and fungal taxonomic dataset to reveal links between taxa and key compounds that may govern DOC concentration. Specifically, we asked the following questions: (1) Do cohorts of high and low DOC samples contain distinct compounds? (2) What compounds or compound classes drive patterns of DOC concentration? and (3) What microbial taxa are associated with DOC concentration and composition? We show that for the microcosms studied, significant positive and negative correlations were identified between microorganisms, chemical compound types, and DOC concentration indicating that certain chemical compound types are linked with increased DOC concentrations and carbon storage potential.

## Materials and Methods

### Experimental Setup

As described in Albright et al. (2020) soil samples were collected from 206 locations throughout the southwestern United States between February and April 2015. Samples were typically collected at locations approximately 80 km apart, at least 15 m from roadways, and from the top 3 cm of the soil surface after removal of surface litter (if any). Samples were collected in sterile 50-ml screw-cap tubes and immediately stored on ice. Samples were stored at 6°C in the laboratory to avoid microbial lysis from freeze-thaw effects and were used within 6 weeks to inoculate microcosms. A map of the sampling locations is published in the Supplementary Material in Albright et al. (2020). Sampling included sites in Texas, Oklahoma, Kansas, Nebraska, Wyoming, Colorado, Utah, New Mexico, and Arizona with grassland-shrub and juniper woodland – grass being the predominant ecosystem types. Sampling was conducted across a wide geographical area to collect a diverse consortium of microbial communities to better power our analysis.

Microcosms were created using 125 mL serum bottles, with each bottle containing approximately 5 g of sand (Accusand; Covia Corp., Ottawa, MN, United States). The microcosms, including 0.02 g of dried pine needles ground in a Wiley Mill (Thomas Scientific, Swedesboro, NJ, United States), were sterilized by autoclaving three times for 1 h each, with at least an 8-h resting interval between each autoclave cycle. Three microcosms per soil sample (*n* = 206 soils, 618 microcosms) each received 1.3 mL of inoculum, pipetted directly onto the initial aliquot of 0.02 g of litter (dried pine needles). The microbial inoculum was extracted from each soil sample (*n* = 206) by suspending 1 g of soil in 9 ml of phosphate-buffered saline (PBS), then generating a 1000-fold dilution in PBS amended with NH_4_NO_3_ at 4.8 mg/ml. The pine needle substrate used in each microcosm was all collected at one location to control for possible differences in the added substrate. Four negative control microcosms, used to confirm the efficacy of sterilization, received equal amounts of PBS and NH_4_NO_3_, but no microbial inoculation. Sealed microcosms were incubated at 25°C in the dark for 14 days to equilibrate the communities, allow the microbes to consume any background DOC provided from the initial soil inoculation, and activate the communities to consume pine needle litter. CO_2_ was evacuated using a vacuum pump and replaced with sterile-filtered air on days 3 and 7. On day 14, an additional 0.1 g of sterilized litter was added to each microcosm and the microcosms were sealed with Teflon-lined crimp caps. The microcosms were incubated at 25°C in the dark for a further 30 days. During this time, CO_2_ was measured by gas chromatography using an Agilent Technologies 490 Micro GC (Santa Clara, CA, United States) on days 2, 5, 9, 16, 23, and 30. After each measurement, the headspace air was evacuated with a vacuum pump and replaced with sterile-filtered air. After the 44-day (total) incubation, microcosms were destructively sampled to measure DOC concentration and characterize DOC chemical and microbial community composition by FTICR-MS and bacterial 16S and fungal 28S rRNA using the Illumina MiSeq sequencer platform, respectively.

### Dissolved Organic Carbon Chemical Composition

Dissolved organic carbon extractions were performed using a rapid, gentle washing procedure to avoid measurement artifacts arising from microbial growth or microbial cell disruption. Specifically, 5 mL of sterile deionized water was added to each microcosm, swirled manually for 30 s, then transferred to two 2-mL microfuge tubes and centrifuged at 16,400 × *g* for 4 min. The supernatants were combined and sterilized by filtration through a 0.2 μm filter. The concentration of DOC in each sample was measured on an OI Analytical model 1010 wet oxidation TOC analyzer (Xylem inc., Rye Brook, NJ, United States), calibrated daily.

A total of 125 DOC samples were selected for chemical composition characterization with a focus on the extremes of DOC concentrations (62 high DOC and 63 low DOC). The chemical composition of DOC was determined using Fourier transform ion cyclotron resonance mass spectrometry (FTICR-MS). FTICR classifies compounds by ratios of C, O, P, N, S, and H which are then used to assign the peak to a compound class; however, specific compound identifications are putative.

For FTICR, the 125 samples consisting of sand and pine litter were sequentially extracted using water, methanol, and chloroform according to Tfaily et al. (2017). The extracts were diluted with methanol to aid in ionization. A 12 Tesla (12T) Bruker SolariX FTICR-MS located at the Environmental Sciences Laboratory in Richland, WA, United States, was used to collect high-resolution mass spectra from each sample. Samples were directly injected into the instrument using a custom automated direct infusion cart operating at 3 μl/min and performed two offline blanks between each sample. The SolariX cart is equipped with an electrospray ionization (ESI) source that was operated in negative ion mode with an applied voltage of −4.2 kV. Ion accumulation time was optimized for each extraction solvent. One hundred and forty-four transients were co-added with a spectral mass window of m/z 100–900, yielding a resolution of 400 K at m/z 381. Spectra were initially recalibrated in the mass domain using homologous series separated by 14 Da (CH_2_ groups). The mass measurement accuracy was typically within 1 ppm for singly charged ions across a broad m/z range (100–900 m/z). Bruker Daltonics DataAnalysis (version 4.2) was used to convert mass spectra to a list of m/z values by applying the FTMS peak picking module with a signal-to-noise ratio (S/N) threshold set to 7 and absolute intensity threshold to the default value of 100. Chemical formulae were assigned using Formularity (Tolić et al., 2017) based on mass measurement error <0.5 ppm, taking into consideration the presence of C, H, O, N, S, and P and excluding other elements. This in-house software was also used to align peaks with a 0.5 ppm threshold. After formula assignment, compounds were categorized into nine classes (Supplementary Figure 1): amino sugar-, carbohydrate-, condensed hydrocarbon-, lignin-, lipid-, protein-, tannin-, unsaturated hydrocarbon-like compounds and unclassified compounds based on their O:C and H:C ratios according to Tfaily et al. (2015, 2017). For each sample, we combined the number of peaks observed from each extraction making sure not to double count chemical formulas to generate the total number of peaks observed. Although these categories are putative, we will drop the “-like” designation throughout this manuscript (e.g., proteins instead of protein-like compounds). We observed an increase in lignin-like compounds between control, high DOC, and low DOC, with the low DOC group exhibiting the greatest number of lignin-like peaks (Supplementary Figure 2A), most likely due to the lack of other compound classes in samples with low DOC, hence enhancing lignin ionization efficiency. Because lignin-like compounds (i.e., phenolic compounds) could only be produced by microbes in response to external stressors, and generally plant-derived lignin declines or transforms during litter degradation, lignin is assumed to be the highest in the control samples. Lignin ionization during ESI appeared to be impacted by the presence of other compounds due to charge competition in the FTICR mass spectrometry measurements. To remove the impact of crowding and improve assessment of compound dynamics, we normalized the number of peaks of each compound class by lignin in each sample (Supplementary Figures 1B, 3). Therefore, lignin served as an internal control. Compound class peak counts were scaled, log-transformed, and mean centered to ensure samples were equally distributed. To identify compound classes that were most likely to underlie DOC concentration, we focused on compound classes that most strongly and positively correlated with DOC concentration as negative correlations between compound classes and DOC concentration may be the result of the crowding effect. To visualize the chemical composition of FTICR compounds in our samples, we used Principal Components Analysis (PCA) and van Krevelen plots. van Krevelen plots illustrate the relationships between the molar ratios of hydrogen to carbon and oxygen to carbon (Van Krevelen, 1950; Wu et al., 2004).

### Microbial Community Composition

Bacterial and fungal composition was characterized as in Albright et al. (2020). Following DOC sampling, material (sand and litter) from each microcosm was frozen at −80°C for DNA extraction. DNA extractions were performed using a DNeasy PowerSoil 96-well plate DNA extraction kit (Qiagen, Hilden, Germany). The standard protocol was used with the following two exceptions: (1) 0.3 grams of material was used per extraction; and (2) bead beating was conducted using a SPEX Certiprep 2000 Geno/Grinder (SPEX SamplePrep, Metuchen, NJ, United States) for 3 min at 1900 strokes/minute. DNA samples were quantified with an Invitrogen Quant-iT™ dsDNA Assay Kit (Thermo Fisher Scientific, Eugene, OR, United States) on a BioTek Synergy HI Hybrid Reader (Winooski, VT, United States). PCR templates were prepared by diluting an aliquot of each DNA stock in sterile water to 1 ng/μl. The bacterial (and archaeal) 16S rRNA gene (V3–V4 region) was amplified using primers 515f-R806 (Bates et al., 2011). The fungal 28S rRNA gene (D2 hypervariable region) was amplified using the LR22R primer (Mueller et al., 2016) and the reverse LR3 primer (Talbot et al., 2014). The 28S rRNA gene sequence is amenable to phylogenetic tree construction and provides genus-level resolution equivalent to that provided by internal transcribed spacer (ITS) sequences (Porras-Alfaro et al., 2014). Preparation for Illumina high-throughput sequencing was undertaken using a two-step approach, similar to that performed by Mueller et al. (2016), with Phusion Hot Start II High Fidelity DNA polymerase (Thermo Fisher Scientific, Vilnius, Lithuania). Amplicons were cleaned using a MoBio UltraClean PCR clean-up kit (Carlsbad, CA, United States), quantified using the same procedure as for the extracted DNA, and then pooled at a concentration of 10 ng each. The pooled samples were further cleaned and concentrated using the MoBio UltraClean PCR clean-up kit. A bioanalyzer was used to assess DNA quality, concentration was verified using qPCR, and paired-end 250 basepair (bp) reads were obtained using an Illumina MiSeq sequencer at Los Alamos National Laboratory. All raw sequence data is available at the National Center for Biotechnology Information (NCBI) Sequence Read Archive (SRA) under accession PRJNA478595.

Bacterial and fungal sequences were merged with PEAR v 9.6 (Zhang et al., 2014), quality filtered to remove sequences with 1% or more low-quality (q20) bases, and demultiplexed using QIIME (Caporaso et al., 2010) allowing no mismatches to the barcode or primer sequence. Sequences with an error rate greater than 0.5 were removed, remaining sequences were dereplicated, singletons were excluded from clustering, OTU clustering was performed at 97% and putative chimeras were identified *de novo* using UCHIME (Edgar, 2013). Bacterial and fungal OTUs were classified using the Ribosomal Database Project (RDP) classifier (Wang et al., 2007). OTUs which were not classified as bacteria or fungi with 100% confidence were removed from the dataset and all OTUs with a phylum classification confidence level of at least 80% were retained. For all lower classifications, a confidence level of at least 70% was used (Tables 1, 2). Confidence scores below 70% are indicated in the table but assignments below 70% confidence are not used for downstream analyses.

**TABLE 1:** Forty-two bacterial taxonomic features consistently associated with DOC concentration in random forest (RF), neural network (NN), and indicator species (IS) analyses (*n* = 349).

OTU^a^	Phyla	Order	Family	Genus	RF Importance	NN Importance	IS stat	IS *P*-value	IS. DOC group
OTU_188	Actinobacteria	Actinomycetales	Nocardioidaceae	Aeromicrobium	0.014	−0.423	0.6	0.03	Low
OTU_179	Actinobacteria	Actinomycetales	Microbacteriaceae	Agromyces (67%)	0.043	−0.457	0.519	0.02	Low
OTU_201	Actinobacteria	Solirubrobacterales	Conexibacteraceae	Conexibacter	0.045	−0.573	0.689	0	Low
OTU_53	Actinobacteria	Actinomycetales	Nocardiaceae	Rhodococcus	0.062	−0.577	0.753	0	Low
OTU_150	Actinobacteria	Actinomycetales	Microbacteriaceae	Subtercola (10%)	0.315	−0.62	0.81	0	Low
OTU_92	Actinobacteria	Actinomycetales	Nocardiaceae	Williamsia	0.164	−0.583	0.766	0	Low
OTU_93	Bacteroidetes	Sphingobacteriales	Chitinophagaceae	Chitinophaga	0.144	−0.591	0.694	0	Low
OTU_125	Bacteroidetes	Flavobacteriales	Flavobacteriaceae	Flavobacterium	0.022	−0.752	0.632	0	Low
OTU_157	Bacteroidetes	Flavobacteriales	Flavobacteriaceae	Moheibacter	0.017	−0.525	0.548	0.015	Low
OTU_249	Bacteroidetes	Sphingobacteriales	Chitinophagaceae	Taibaiella	0.015	−0.644	0.556	0.02	Low
OTU_107	Firmicutes	Bacillales	Paenibacillaceae	Paenibacillus	0.018	−0.441	0.697	0	Low
OTU_210	Planctomycetes	Planctomycetales	Planctomycetaceae	Pirellula (44%)	0.012	−0.71	0.55	0	Low
OTU_3	Proteobacteria	Burkholderiales	Alcaligenaceae	Achromobacter	0.045	−0.591	0.738	0	Low
OTU_12	Proteobacteria	Caulobacterales	Caulobacteraceae	Caulobacter	0.316	−0.624	0.769	0	Low
OTU_2512	Proteobacteria	Pseudomonadales	Pseudomonadaceae	Cellvibrio	0.023	−0.448	0.416	0.01	Low
OTU_96	Proteobacteria	Pseudomonadales	Pseudomonadaceae	Cellvibrio	0.017	−0.462	0.68	0	Low
OTU_55	Proteobacteria	Rhizobiales	Hyphomicrobiaceae	Devosia	1	−1	0.819	0	Low
OTU_574	Proteobacteria	Rhizobiales	Hyphomicrobiaceae	Devosia	0.055	−0.462	0.763	0	Low
OTU_379	Proteobacteria	Gammaproteobacteria	Halioglobus (51%)	Halioglobus (51%)	0.023	−0.673	0.598	0	Low
OTU_70	Proteobacteria	Xanthomonadales	Xanthomonadaceae	Luteimonas	0.024	0.515	0.563	0	High
OTU_1452	Proteobacteria	Rhizobiales	Aurantimonadaceae	Martelella (61%)	0.123	−0.595	0.697	0	Low
OTU_40	Proteobacteria	Burkholderiales	Oxalobacteraceae	Massilia	0.033	−0.463	0.728	0.03	Low
OTU_129	Proteobacteria	Rhizobiales	Methylobacteriaceae	Methylobacterium	0.018	0.46	0.551	0	High
OTU_224	Proteobacteria	Rhizobiales	Bradyrhizobiaceae (62%)	Nitrobacter (23%)	0.067	−0.774	0.689	0	Low
OTU_78	Proteobacteria	Rhizobiales	Bradyrhizobiaceae	Nitrobacter (47%)	0.018	−0.596	0.613	0	Low
OTU_85	Proteobacteria	Sphingomonadales	Sphingomonadaceae	Novosphingobium	0.04	−0.636	0.686	0	Low
OTU_39	Proteobacteria	Bdellovibrionales	Bacteriovoracaceae	Peredibacter	0.033	−0.756	0.723	0	Low
OTU_22	Proteobacteria	Rhizobiales	Phyllobacteriaceae	Phyllobacterium	0.032	0.509	0.729	0.04	High
OTU_3824	Proteobacteria	Rhizobiales	Brucellaceae	Pseudochrobactrum	0.014	−0.561	0.552	0.03	Low
OTU_572	Proteobacteria	Burkholderiales	Oxalobacteraceae	Pseudoduganella	0.013	−0.495	0.411	0	Low
OTU_122	Proteobacteria	Rhizobiales	Xanthobacteraceae (65%)	Pseudolabrys (63%)	0.06	−0.848	0.781	0	Low
OTU_1	Proteobacteria	Pseudomonadales	Pseudomonadaceae	Pseudomonas	0.038	0.475	0.8	0	High
OTU_3002	Proteobacteria	Pseudomonadales	Pseudomonadaceae	Pseudomonas (37%)	0.014	−0.432	0.638	0.005	Low
OTU_11	Proteobacteria	Rhizobiales	Rhizobiaceae	Rhizobium	0.022	−0.85	0.805	0	Low
OTU_6	Proteobacteria	Rhizobiales	Rhizobiaceae	Rhizobium	0.48	−0.75	0.852	0	Low
OTU_273	Proteobacteria	Myxococcales	Sandaracinaceae	Sandaracinus	0.023	−0.689	0.597	0.02	Low
OTU_29	Proteobacteria	Rhodospirillales	Rhodospirillaceae	Skermanella	0.046	0.592	0.778	0	High
OTU_99	Proteobacteria	Rhizobiales	Rhizobiales_incertae_sedis (27%)	Variibacter (22%)	0.016	−0.628	0.763	0	Low
OTU_109	Verrucomicrobia	Verrucomicrobiales	Verrucomicrobiaceae	Luteolibacter	0.037	−0.57	0.661	0.01	Low
OTU_34	Verrucomicrobia	Verrucomicrobiales	Verrucomicrobiaceae	Luteolibacter	0.056	−0.814	0.813	0	Low
OTU_397	Verrucomicrobia	Opitutales	Opitutaceae	Opitutus	0.031	−0.772	0.622	0	Low
OTU_198	Verrucomicrobia	Verrucomicrobiales	Verrucomicrobiaceae	Roseimicrobium	0.019	−0.718	0.673	0	Low

*^a^A confidence score cutoff of 70% was used for taxonomic assignment for each OTU.*

*Confidence scores below the cutoff are given in parentheses. The lowest taxonomic level above the 70% cutoff score was used for all downstream analyses.*

**TABLE 2:** Nine fungal taxonomic features consistently associated with DOC concentration were determined from random forest (RF), neural network (NN), and indicator species (IS) analyses.

OTU*^a^*	Phyla	Order	Family	Genus	RF importance	NN importance	IS stat	IS *P*-value	IS DOC group
OTU_18	Ascomycota	Eurotiales	Trichocomaceae	*Aspergillus*	0.491	0.734	0.686	0	High
OTU_1	Ascomycota	Eurotiales	Trichocomaceae	*Emericella* (56%)	1	−0.766	0.778	0.02	Low
OTU_780	Ascomycota	Eurotiales	Trichocomaceae	*Emericella*	0.62	−0.748	0.722	0	Low
OTU_20	Ascomycota	Hypocreales	Nectriaceae	*Gibberella*	0.405	0.876	0.763	0.01	High
OTU_325	Ascomycota	Hypocreales	Nectriaceae	*Gibberella*	0.238	0.741	0.664	0	High
OTU_1212	Ascomycota	Pleosporales	Pleosporaceae	*Alternaria*	0.162	1	0.603	0.015	High
OTU_7	Ascomycota	Pleosporales	Pleosporaceae	*Alternaria*	0.842	0.764	0.758	0.04	High
OTU_24	Ascomycota	Pleosporales	Sporormiaceae	*Preussia* (68%)	0.362	0.963	0.559	0	High
OTU_178	Ascomycota	Incertae sedis	Batistiaceae	*Batistia*	0.157	−0.783	0.468	0	Low

*^a^A confidence score cutoff of 70% was used for taxonomic assignment for each OTU. Confidence scores below the cutoff are given in parentheses. The lowest taxonomic level above the 70% cutoff score was used for all downstream analyses.*

*We used a confidence score cutoff of 70% for taxonomic assignment for each OTU and percentages are given in parentheses for confidence levels below the cutoff. We used the lowest taxonomic level that was above the 70% cutoff score for all downstream analyses (n = 377).*

Following quality control and classification, 9,576,525 sequences from 349 of the original 618 microcosms were obtained for bacteria and 13,124,107 sequences from 377 microcosms were obtained for fungi. These sorted into 2,527 OTUs for bacteria (an average of 275 per microcosm, SE = 8) and 753 OTUs for fungi (an average of 47 per microcosm, SE = 1). For all analyses, bacterial communities were rarefied to 1,023 sequences while fungal communities were rarefied to 2,032 sequences.

### Identifying Key Microbial Taxa

To determine bacterial and fungal taxa significantly associated with DOC concentration, we used two machine learning techniques (i.e., random forests and neural networks) combined with conventional indicator species analysis (Thompson et al., 2019). Taxa jointly identified by all three methods were selected as the most robust predictive features.

For random forest feature selection, we used the random forest regressor made available by Scikit-learn (Pedregosa et al., 2011). To identify taxa, we performed feature ranking that randomly samples 80% of the training data over 50 iterations and the resulting taxa with the highest average feature ranking values over all iterations were identified. For neural network analysis, we built a feed-forward neural network using Theano (Al-Rfou et al., 2016) and Python 3.7 with a randomized search algorithm for determining model hyper-parameters implemented with Scikit-learn (Pedregosa et al., 2011). We used the default single hidden layer with 15 nodes with sigmoid activation functions and a single output layer with linear activation function. We used the randomized hyper-parameter search to find the optimum hidden layer size, learning rate, and regularization coefficient using mean squared error as the cost function. Once the cost function applied to the test data fails to decrease over ten training iterations, training stops. We performed indicator species analysis in Python 3.7 with the methods described in Dufrêne and Legendre (1997) using a 95% confidence cutoff.

### Statistics

To identify compound classes significantly associated with DOC concentration, Spearman correlation analysis was used to determine significant correlations (*P* < 0.05) between FTICR compound classes based on the number of peaks assigned to each compound class (not the summed intensity) and DOC concentration. Spearman correlation analysis was also used to determine bacterial and fungal taxa most strongly associated with those compound classes. The network diagram summarizing Spearman correlations between taxa, metabolites, and DOC was created using Cytoscape based on *R*-values from Spearman correlation analysis (Shannon et al., 2003). PERMANOVA and ANOSIM from the vegan package (Oksanen et al., 2020) in R were used to determine if the composition of FTICR compounds in the high and low DOC cohorts were significantly different. Unless otherwise mentioned, all statistical analysis was done in R (R Core Team, 2021) and figures were created using the package ggplot2 (Wickham, 2016).

## Results

### Dissolved Organic Carbon Chemical Composition Differs Between High and Low Dissolved Organic Carbon Cohorts

**FIGURE 1:**
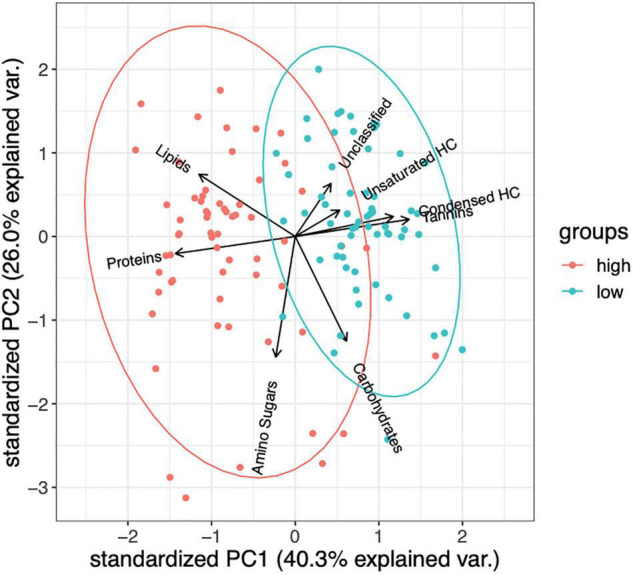
Biplot analysis indicates that high and low DOC groups are enriched with different FTICR compound classes (PERMANOVA, *F* = 46.46, *R*^2^ = 0.27, *P* = 0.001; ANOSIM, *R* = 0.45, *P* = 0.001). Ellipses indicate 95% confidence intervals (*n* = 125 samples).

**FIGURE 2:**
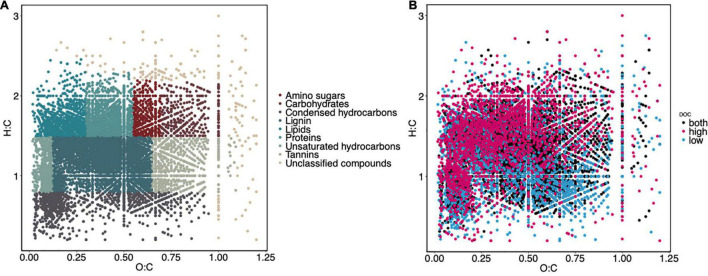
The DOC pool is chemically diverse, and the high and low DOC groups contain distinct compounds. Van Krevelen plots of compounds found in all samples categorized by compound class **(A)** and compounds found in high DOC, low DOC, and both DOC groups **(B)**.

The only lignin inputs were from the starting pine needle substrate, thus we used lignin as an internal control to better compare the other changes in chemical compounds between controls (no microbial inoculation), and high and low DOC samples (Supplementary Figures 1B, 3). High and low DOC cohorts contained distinct compounds as detected by FTICR analysis (PERMANOVA, *F* = 46.46, *R*^2^ = 0.27, *P* = 0.001; ANOSIM, *R* = 0.45, *P* = 0.001; Figures 1, 2). Biplot analysis indicated that the high DOC cohort was associated with proteins, lipids, and amino sugars while compounds associated with low DOC included unsaturated and condensed hydrocarbons, tannins, and unclassified compounds (Figure 1). Carbohydrate abundance did not appear to associate with high or low DOC (Figure 1 and Supplementary Figures 1, 2).

### Fourier Transform Ion Cyclotron Resonance Compounds Correlate With Dissolved Organic Carbon

**FIGURE 3:**
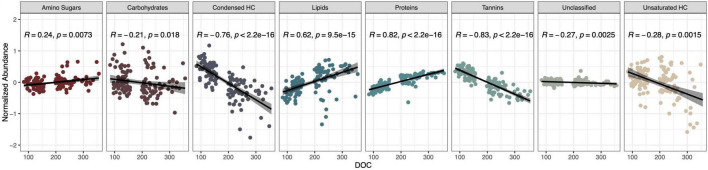
Proteins and tannins are the compound classes most strongly correlated with DOC concentration. Spearman correlation coefficients (*R*), *R*^2^-values, and *p*-values are shown.

Dissolved organic carbon concentration significantly correlated with all compound classes (Spearman correlation, *P* < 0.05, Figure 3), and most strongly with tannins (*R* = −0.83) and proteins (*R* = 0.82). Proteins and lipids showed strong positive correlations with DOC concentration while tannins, condensed hydrocarbons, and unsaturated hydrocarbons negatively correlated with DOC concentration (Figure 3). Further, amino sugars, carbohydrates, and unclassified compounds showed weak albeit significant correlations (Figure 3). Proteins also correlated with the fraction of DOC binding to the common soil mineral, aluminum oxide (*R* = 0.29; Supplementary Figure 4).

### Microbial Taxa Governing Dissolved Organic Carbon

To examine microbial taxa most closely linked to DOC concentration, we used two machine learning methods (random forest and neural network) and indicator species analysis. We identified 42 of 2,527 bacterial OTUs and 9 of 753 fungal OTUs associated with DOC concentration (Tables 1, 2). Proteobacteria made up the majority of bacterial OTU associations with 26 of the 42 OTUs (Table 1). The 9 fungal OTUs were all Ascomycota with Eurotiales (3/9) and Pleosporales (3/9) being the most represented orders associated with DOC concentration (Table 2).

**FIGURE 4:**
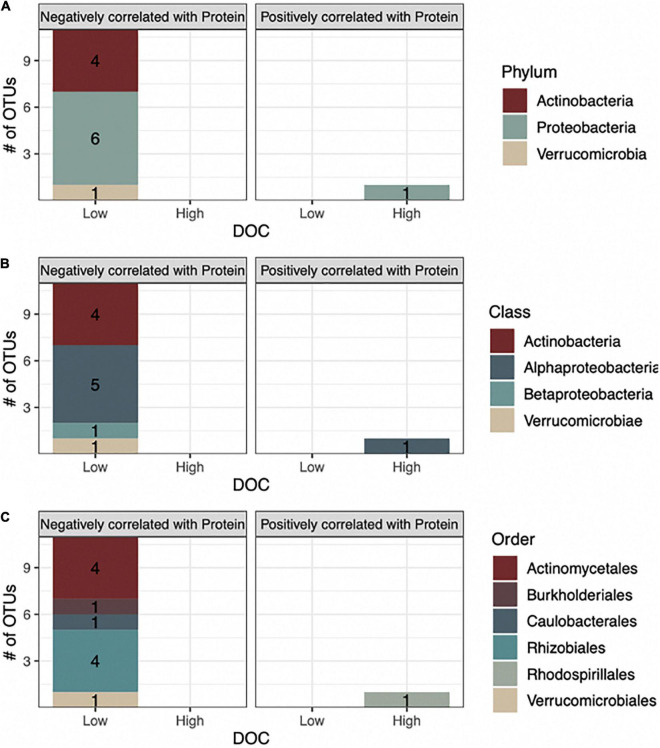
The majority of bacterial OTUs that are associated with high or low DOC are negatively correlated with protein. Stacked barplots indicate the number of bacterial OTUs that correlated negatively (left barplots) or positively with protein (right barplots) and their association with either high or low DOC based on indicator species analysis is shown at **(A)** phyla, **(B)** class, and **(C)** order levels.

**FIGURE 5:**
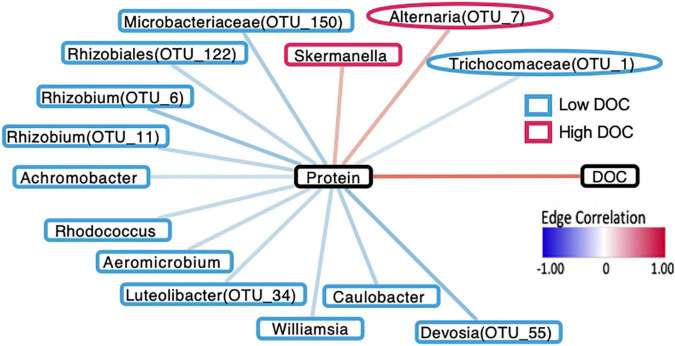
Network diagram demonstrating that interactions between microbial taxa, proteins, and DOC are in accordance with the machine-learning assignments for microorganisms in terms of DOC concentration. Borders around taxa indicate whether they are associated with high DOC (red) or low DOC (blue). Bacterial taxa are indicated by rectangular borders and fungal taxa are indicated by oval borders. Black borders indicate DOC and protein compounds.

To link microbial taxa with chemical composition, we focused on microbial associations with the protein compound class because it had the strongest positive correlation with DOC concentration and is associated with microbial activity (*R* = 0.82, *P* < 0.01, Figure 3). In contrast, tannins, the strongest negatively correlated metabolite with DOC concentration (*R* = −0.83, *P* < 0.01), are generally associated with plant production (Mutabaruka et al., 2007). We performed Spearman correlations on the 42 taxa identified through machine learning and found that 12 bacterial OTUs significantly correlated with protein abundance (*P* < 0.05, Figure 4). Likewise, 2 of the 9-machine learning-selected fungal features correlated significantly with protein abundance (Figure 5). 92% of the 12 bacterial OTUs (11/12) negatively correlated with protein. At the genus level, *Skermanella* (Rhodospirillaceae, Rhodospirillales) (*R* = 0.41, *P* < 0.01) was the only taxa positively correlated with protein abundance, while *Devosia* (Hyphomicrobiaceae, Rhizobiales, OTU 55) (*R* = −0.56, *P* < 0.01) and *Rhizobium* (Rhizobiaceae, Rhizobiales, OTU 6) (*R* = −0.49, *P* < 0.01) were most negatively correlated with protein (Figure 5). For fungi, *Alternaria* (Pleosporaceae, Pleosporales, OTU 7) positively correlated with protein (*R* = 0.44, *P* = 0.01), while the Trichocomaceae family (Eurotiales, OTU 1) negatively correlated with protein (*R* = −0.20, *P* = 0.01) (Figure 5).

In linking taxa-protein correlations with DOC concentration, we found that all 11 of the bacterial OTUs negatively correlated with protein were classified as being more abundant in low DOC, while the lone bacterial OTU positively related to protein was more abundant in high DOC (Figures 4, 5). Following the same trend, the fungal OTU positively correlated with protein was associated with high DOC and the fungal OTU negatively correlated with protein was associated with low DOC. The 11 bacterial taxa negatively correlated with protein represent 3 different phyla and 5 different orders, while the positively correlated taxon belongs to the order *Rhodospirillales* in the phylum Proteobacteria (Figure 4).

We used correlational analyses to determine if machine learning assignments of taxa to high and low DOC were consistent with the direction and magnitude of taxa-compound correlations and compound-DOC correlations (Figure 5). All of the 14 bacterial or fungal taxa that had significant correlations to protein were consistent with their DOC concentration assignment (Figure 5). For example, Microbacteriaceae (OTU 150) was associated with low DOC and correlated negatively with protein (positively correlated with DOC).

## Discussion

Determining how metabolite composition correlates with DOC concentration is an important step in understanding the mechanisms of microbially mediated C sequestration. The chemical composition of DOC plays an important role in C fate because compounds can vary in their recalcitrance and capacity to bind to soil minerals, impacting microbial accessibility. To understand the molecular basis of this relationship while also gaining insight into key physiological processes driving DOC concentration, we analyzed DOC samples from laboratory microcosms representing microbial community cohorts that exhibit extreme differences in C fate (i.e., low and high DOC). Our novel multi-pronged approach employed detailed compound characterization (FTICR), machine learning, and indicator species analysis to provide evidence that: (1) high and low DOC microcosms contain different compounds, (2) protein-like compounds and tannin-like compounds most strongly correlated with DOC concentration (although these categories are putative we will drop the “-like” designation), and (3) 51 key microbial taxa are associated with DOC concentration and may influence DOC concentration through association with specific metabolites. Our study fills a major gap by identifying connections between soil metabolites, microorganisms, and C flow during the early phase of litter decomposition. Establishing these relationships is foundational to improve modeling of C flow and manipulation of C storage.

### Dissolved Organic Carbon Composition Is Linked With Dissolved Organic Carbon Concentration

Dissolved organic carbon composition of the high and low DOC cohorts was significantly different, indicating that certain compounds may govern DOC concentration and carbon flow. Of the eight compound classes identified by FTICR, proteins and tannins had the highest correlations to DOC concentration. Proteins were positively correlated with DOC concentration in agreement with previous work (Kallenbach et al., 2016), which may be due to their tendency to complex with soil organic and inorganic molecules (Hsu and Hatcher, 2005). Alternatively, proteins may correlate with increased DOC since an increase in enzymes involved with degradation will result in more primary decomposition of plant litter (Datta et al., 2017). The protein portion of DOC may originate from the plant litter, microbial necromass (Navarre and Schneewind, 1999), or from enzymes secreted by microbes into the extracellular matrix for decomposition (Schneider et al., 2012; Baldrian, 2017). As decomposition of plant litter progresses, a greater proportion of protein in dissolved organic matter (DOM) is reported to be microbially derived (Schulze, 2005; Schneider et al., 2012), rather than plant-derived, perhaps because most of the plant protein is consumed in the early phases of litter decomposition. Indeed, the relative contribution of plant-derived proteins has been observed to decline with increasing soil depth while the proportion of microbial-derived proteins increases (Schulze, 2005). Tannins, on the other hand, had a negative correlation with DOC levels. Tannins are complex compounds produced by plants that have been assumed to be resistant to degradation due to their polyphenolic nature (Mutabaruka et al., 2007). As such, a positive correlation between tannins and DOC concentration is expected, contrary to our observation. Alternatively, tannins could correlate negatively with DOC concentration by reducing decomposition rates. Tannins can inactivate enzymes either through complexation or by acting as redox-buffers which may neutralize the oxidative enzymes necessary for breaking down reduced compounds such as lignin (Triebwasser et al., 2012). Condensed hydrocarbons are another compound class considered to be recalcitrant, and a similar negative correlation with DOC was observed instead of the expected positive correlation (Figure 3). Since FTICR data results in relative values, condensed hydrocarbons and tannins may be more detectable in low DOC samples as a result of decreased levels of other compounds that would obscure detection. Thus, these negative correlations may be due to negative selection where other compounds are depleted or absent in low DOC samples resulting in higher relative concentrations of tannins and condensed hydrocarbons (Breitling et al., 2006). Thus, negative correlations of tannins and condensed hydrocarbons with DOC may be good metrics for predicting C fate when using relative abundances but may not hold true when measuring absolute abundances.

### Microbial Taxa Governed Dissolved Organic Carbon Composition

Using machine learning and indicator species analyses we were able to identify 51 taxa associated with DOC abundance from an initial pool of 3,280 different taxa (2,527 bacterial taxa and 753 fungal taxa) across hundreds of samples over a large geographic area. To gain insight to potential mechanisms of C flow we identified correlations between the identified taxa and metabolites. We focused on protein, because it was the compound class most significantly and positively associated with DOC concentration. Positive and negative correlations between taxa and metabolites have been used to indicate: (1) microbial production or consumption of metabolites (Swenson et al., 2018), which is determined by microbial substrate preference (Banerjee et al., 2016; Zhalnina et al., 2018), (2) taxa that may suppress other microbes that consume or produce protein, and/or (3) taxa that may thrive in the presence of high or low protein abundance. To assess distinguishing characteristics of taxa linked to high versus low DOC, we examined characteristics at multiple taxonomic levels because more information exists at broader taxonomic levels and many traits are conserved within related taxa (Martiny et al., 2013; Morrissey et al., 2016).

A large majority (92%) of the bacterial OTUs that correlated with protein had a negative correlation, suggesting that the microbial association to protein abundance in DOC may be primarily driven by protein consumption. Protein is a good microbial source of C and N and in the short-term may be consumed by bacteria before becoming inaccessible to microbes when it binds to organic and mineral compounds (Gibbs and Barraclough, 1998). The 11 taxa that correlated negatively with protein represented 3 phyla, 4 classes, and 5 orders, indicating that protein consumption is a generalist trait rather than specific to a certain clade. Only 1 taxon, belonging to the phylum Proteobacteria, had a positive correlation with protein. This is consistent with studies of plant litter decomposition that track soil protein to its source organism. Schneider et al. (2012) found that 59–90% of litter degrading bacterial protein in soil originated from Proteobacteria depending on site, while Liu et al. (2015) found that Proteobacteria generated 56% of all bacterial protein present in soil communities. More generally, Proteobacteria are copiotrophic and have a propensity to dominate microbial communities in the presence of labile organic substrates which are available during the early stages of degradation (Fierer et al., 2007; Keiblinger et al., 2012; Degrune et al., 2017). For fungi, the two OTUs that correlated with protein belong to Ascomycota, a phylum of dominant cellulose decomposers (Stursová et al., 2012; Wilhelm et al., 2017). Similar to Proteobacteria, Ascomycota tend to produce a majority of the fungal protein in plant litter decomposition studies, producing >80% of fungal protein in one study (Schneider et al., 2012). The significant correlations of Ascomycota OTUs, and not Basidiomycota, another dominant decomposer, may be because Ascomycota are prominent in early stages of decomposition (Wilhelm et al., 2017).

The identification of microbes associated with DOC concentration was performed using random forest, neural network, and indicator species analyses. Machine learning techniques are able to identify complex relationships and not just linear correlations (Thompson et al., 2019). However, the criteria used by the machine learning method for feature selection are not always readily apparent because algorithms incorporate many factors that may be hidden behind layers that are difficult to interpret (Ghannam and Techtmann, 2021). By incorporating correlations between taxa, proteins, and DOC, we were able to identify possible interactions underlying the machine learning assignments. For example, taxa assigned to high and low DOC with machine learning methods showed consistent correlations with protein compounds (Figures 4, 5). Correlation with the protein compound class proved to be a consistent predictor of taxa association to high or low DOC. Combining metabolite correlations with machine learning predictions of taxa driving DOC abundance illuminates physiological processes (e.g., extracellular protein consumption and production) that underpin C flow.

## Conclusion

We found that DOC concentration during the early stages of pine litter decomposition is linked to changes in DOC composition. In a first step toward mechanism, we linked microbial taxa with metabolite abundance by determining the direction and magnitude of significant correlations to metabolite compound classes and their association with high and low DOC. Our results indicate that protein-like compounds are positively correlated with DOC while tannin-like compounds are negatively correlated with DOC. Further, the high proportion of taxa negatively correlated with proteins suggest that microbial consumption of protein is likely a significant driver of DOC concentration. Future studies are warranted to directly test the impact of individual and groups of taxa on the concentration, consumption, and production of DOC metabolites to improve efforts to model C flow and potentially manipulate C flow with microbes.

## Data Availability Statement

The datasets presented in this study can be found in online repositories. The names of the repository/repositories and accession number(s) can be found below: https://massive.ucsd.edu/ProteoSAFe/private-dataset.jsp?task=fd35e221dc0240ffabd418428413662b, MSV000088109.

## Author Contributions

JD, MA, and MT conceptualized and planned the study. DU, MA, and JD performed the experiments. TC, DU, JTo, JTh, BM, MA, and MT performed the analyses. TC, DU, MA, VB, and JD were involved in writing the manuscript. All authors contributed to the article and approved the submitted version.

## Conflict of Interest

The authors declare that the research was conducted in the absence of any commercial or financial relationships that could be construed as a potential conflict of interest.

## Publisher’s Note

All claims expressed in this article are solely those of the authors and do not necessarily represent those of their affiliated organizations, or those of the publisher, the editors and the reviewers. Any product that may be evaluated in this article, or claim that may be made by its manufacturer, is not guaranteed or endorsed by the publisher.
