# Supplementary material for: Microbial Communities Influence Soil Dissolved Organic Carbon Concentration by Altering Metabolite Composition
Source: Front Microbiol. 2022 Jan 20;12:799014. doi: 10.3389/fmicb.2021.799014 (PMC8811196; doi:10.3389/fmicb.2021.799014)
Supplement: Supplementary file 1 [file Presentation_1.pdf]

SUPPLEMENTARY INFORMATION

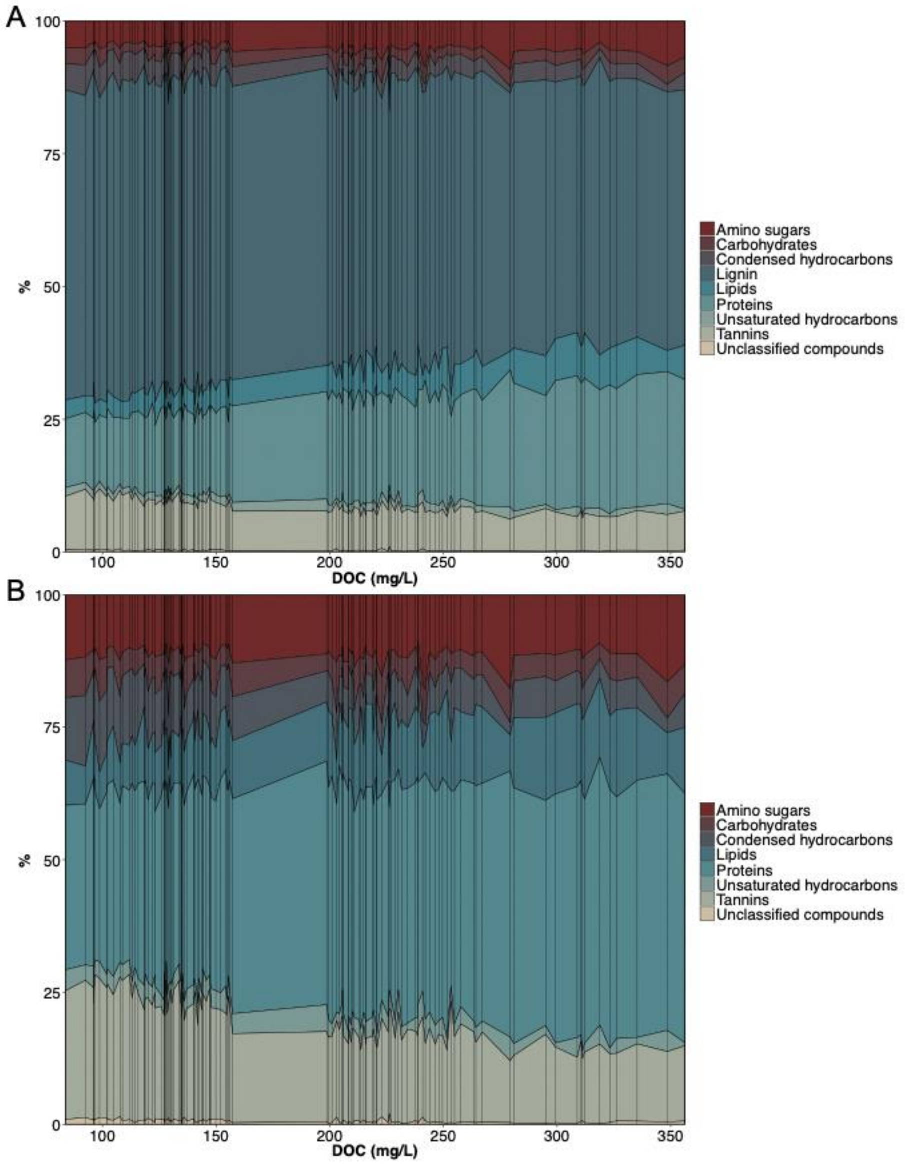

Supplementary Figure 1. Relative abundance (%) of the raw number of peaks in each compound class (A) and the number of peaks in each compound class normalized by lignin (B) in each DOC sample (n=125). Each vertical line represents a sample.

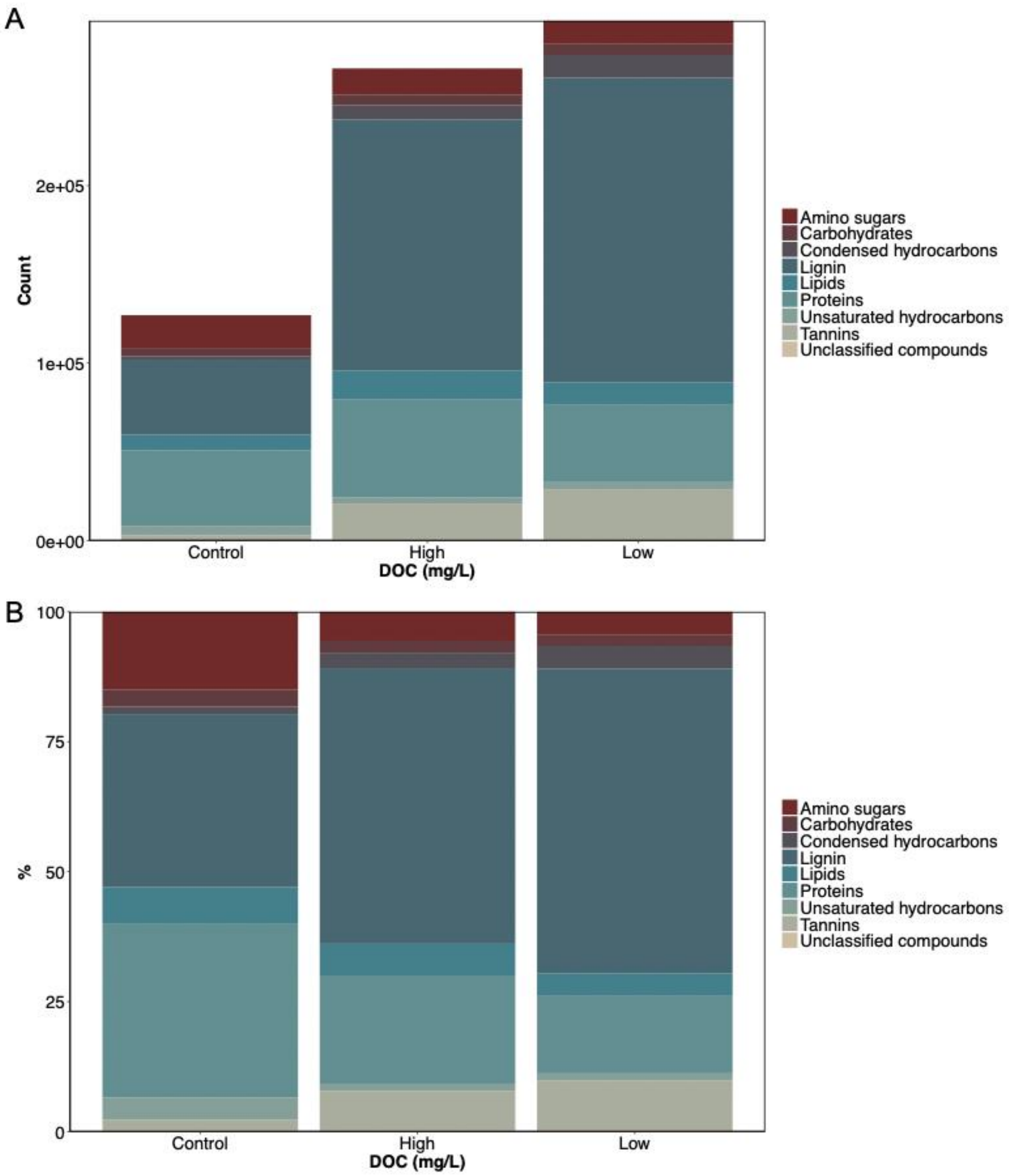

Supplementary Figure 2. Raw number of peaks (A) and relative abundance (B) of each compound class for the control, high, and low DOC groups (n=125 samples and 4 controls).

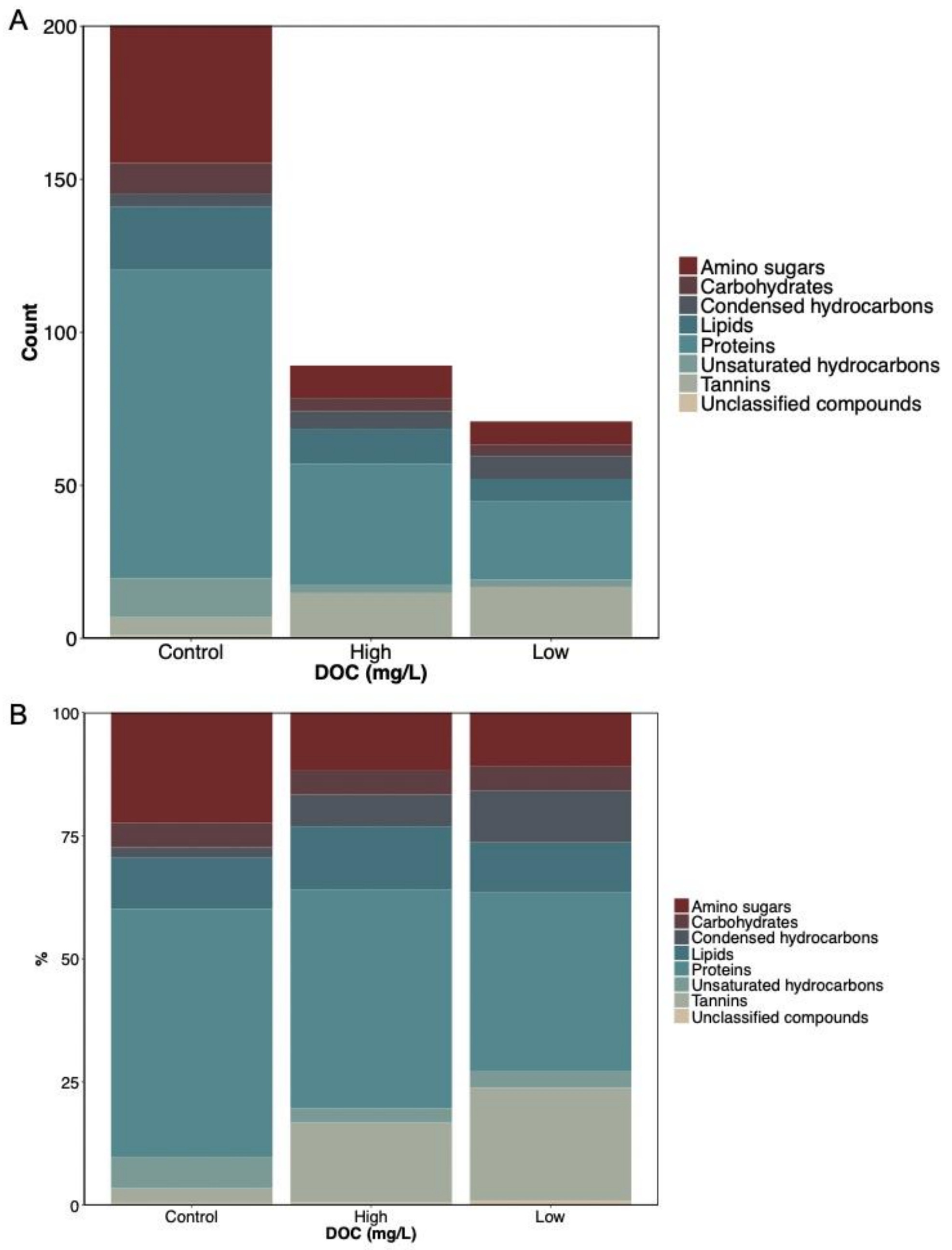

Supplementary Figure 3. Raw number of peaks (A) and relative abundance (B) of each compound class normalized by lignin in each sample for the control, high, and low DOC groups (n=125 samples and 4 controls)

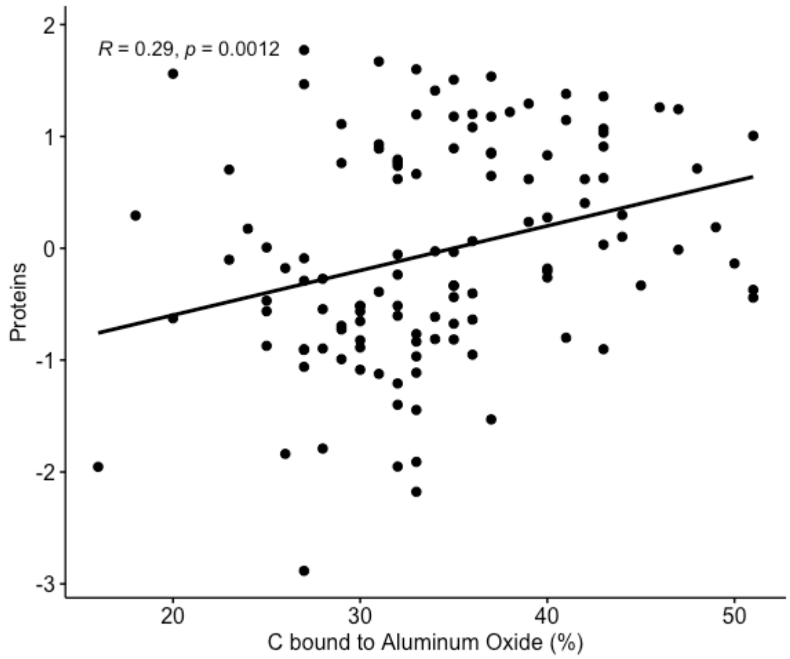

Supplementary Figure 4. Proteins correlate with fraction of C bound to aluminum oxide. Pearson correlation coefficient (*R*) and *P*-value are shown.
